# Leaching and degradation of ^13^C_2_-^15^N-glyphosate in field lysimeters

**DOI:** 10.1007/s10661-019-8045-4

**Published:** 2020-01-21

**Authors:** Peter Gros, Ralph Meissner, Marisa A. Wirth, Marion Kanwischer, Holger Rupp, Detlef E. Schulz-Bull, Peter Leinweber

**Affiliations:** 1 Agricultural and Environmental Science, Soil Science, University of Rostock, Justus-von-Liebig-Weg 6, 18051 Rostock, Germany; 2 Department of Soil System Science, Helmholtz Centre for Environmental Research, Lysimeter Station, Falkenberg 55, 39615 Altmärkische Wische, Germany; 3 Department of Marine Chemistry, Leibniz Institute for Baltic Sea Research Warnemünde, Seestrasse 15, 18119 Rostock, Germany

**Keywords:** Pesticide, Fate, IR-MS, HPLC-MS/MS, Stable isotopes, Environmental detection

## Abstract

Glyphosate (GLYP), the globally most important herbicide, may have effects in various compartments of the environment such as soil and water. Although laboratory studies showed fast microbial degradation and a low leaching potential, it is often detected in various environmental compartments, but pathways are unknown. Therefore, the objective was to study GLYP leaching and transformations in a lysimeter field experiment over a study period of one hydrological year using non-radioactive ^13^C_2_-^15^N-GLYP labelling and maize cultivation. ^15^N and ^13^C were selectively measured using isotopic ratio mass spectrometry (IR-MS) in leachates, soil, and plant material. Additionally, HPLC coupled to tandem mass spectrometry (HPLC-MS/MS) was used for quantitation of GLYP and its main degradation product aminomethylphosphonic acid (AMPA) in different environmental compartments (leachates and soil). Results show low recoveries for GLYP (< 3%) and AMPA (< level of detection) in soil after the study period, whereas recoveries of ^15^N (11–19%) and ^13^C (23–54%) were higher. Time independent enrichment of ^15^N and ^13^C and the absence of GLYP and AMPA in leachates indicated further degradation. ^15^N was enriched in all compartments of maize plants (roots, shoots, and cobs). ^13^C was only enriched in roots. Results confirmed rapid degradation to further degradation products, e.g., ^15^NH_4_^+^, which plausibly was taken up as nutrient by plants. Due to the discrepancy of low GLYP and AMPA concentrations in soil, but higher values for ^15^N and ^13^C after the study period, it cannot be excluded that non-extractable residues of GLYP remained and accumulated in soil.

**Electronic supplementary material:**

The online version of this article (10.1007/s10661-019-8045-4) contains supplementary material, which is available to authorized users.

## Introduction

Glyphosate (GLYP) is an important herbicide in the world, annually > 1 million tonnes are applied (Richmond 2018). GLYP reaches the soil either by direct spraying or indirectly by release through plant roots (Neumann et al. 2006; Laitinen et al. 2007). Rapid microbial degradation has been reported in soil, leading to the most predominant degradation product aminomethylphosphonic acid (AMPA). This pathway has been documented extensively and was reviewed by Borggaard and Gimsing (2008). Degradation products can further react to CO_2_ and NH_4_^+^. Degradation rates for GLYP vary significantly and half-life values from 2 to 180 days have been reported (Borggaard and Gimsing 2008; Tang et al. 2019). GLYP can interact strongly with organic and inorganic molecules at a variety of binding sites such as (i) the soil organic matter, e.g., peptides, carbohydrates, or phenolic structures (Gros et al. 2017; Ahmed et al. 2018) or (ii) mineral surfaces, e.g., goethite or montmorillonite, as has been demonstrated in laboratory experiments and quantum chemical modeling (Morillo et al. 1997; Ahmed et al. 2017). These interactions lead to strong and high sorption, as has been shown in laboratory batch sorption experiments (Dion et al. 2001; Okada et al. 2016; Gros et al. 2017). Although strong sorption and degradation are antagonistic effects, both are supported under normal or low tillage soil management and should prevent GLYP from distributing through soil (Kjær et al. 2005; Bergström et al. 2011). Contrary to this assumption, GLYP frequently has been detected in ground and surface water (Coupe et al. 2012) even above the regulatory limit of 0.1 μg L^−1^ in the EU (Van Stempvoort et al. 2014; Skeff et al. 2015). The mechanisms of GLYP translocation from place of application through the drainage system into waterways and estuaries are still unclear but need to be understood for preventing these undesired translocations.

Lysimeters are suitable research facilities that enable monitoring and assessing of nutrient or pollutant balances in disturbed or undisturbed soil columns (Führ et al. 1998). Only a few field lysimeter experiments have been conducted with non-labeled GLYP (Malone et al. 2004), where leachate waters were analyzed through fluorescence detection after derivatization of the analyte. In the laboratory, lysimeter experiments with GLYP often have been conducted using non-stable, radioactive ^14^C isotopic labelling, which provides advantages of sensitive quantitation and recovery by scintillation (e.g., Al-Rajab et al. 2008). Safety restrictions for the use of radioactively labeled substances make it necessary to evaluate experimental data from laboratory experiments under more practically relevant field conditions. Utilization of ^13^C_2_-^15^N-GLYP (GLYP*i*), which contains stable isotopes, combines the advantages of non-radioactive GLYP for field studies with the sensitivity of labelling (in analogy to ^14^C-GLYP) for environmental monitoring in laboratory experiments (Muskus et al. 2019). Using that non-radioactively labeled GLYP*i* enables the detection using isotopic ratio mass spectrometry (IR-MS) complementary to high performance liquid chromatography coupled to electrospray ionization mass spectrometry (HPLC-ESI-MS/MS) for environmental measurements of extractable residues of GLYP*i* and AMPA*i*. This experimental approach has not been applied in field lysimeter studies so far. Therefore, we hypothesize that the methodological approach with GLYP*i*, IR-MS, and HPLC-ESI-MS/MS enables to study the fate and possible translocation of GLYP under field conditions.

## Material and methods

The leaching experiment was set up in two field lysimeters (non-weighing zero tension), which were installed in the Lysimeter Station at the Helmholtz Centre for Environmental Research-UFZ (Falkenberg, Germany; 52°51′ N, 11°48′ E). These lysimeters were constructed in 1981 in sheet steel vessels with cuboid shape of 1 × 1 m surface area and 1.25 m depth. The lysimeters were filled with sandy loam (0–30 cm topsoil: 74% sand, 14% silt, 12% clay, pH 4.8, organic C = 1.1%; 30–100 cm subsoil: 75% sand, 17% silt, 8% clay, pH 5.6, organic C = 0.2%) and an additional 25 cm-drainage layer composed of three sublayers (sand, gravel, and coarse gravel) at the bottom. The soil texture is representative for the river Elbe valley in the Federal State Saxony-Anhalt. Conventional agricultural management was oriented according to best management practice. In 2017, maize was planted which was embedded in a regionally typical crop rotation of sugar beets-winter wheat-potatoes-winter barley-maize. Details on the lysimeter site and management history have been published previously (Meissner et al. 2010; Rupp et al. 2018). The present study investigated a period of one hydrological year starting in the hydrological summer semester in May 2017. Any weeds were removed mechanically, followed by GLYP*i* application (2017/24/04) via spraying as a worst-case scenario. Application rate was equivalent to maximum allowed annual for Germany (3.6 kg ha^−1^ a^−1^) with practical concentration of GLYP formulations (480 g kg^−1^) (360 mL GLYP*i*, dissolved in 750 mL H_2_O). Drift by air flow was prevented by temporally fencing the application area with a ring of steel (1 m in height). Three days after GLYP*i* application, 5 L of the conservative KBr tracer solution was applied at a rate corresponding to 40 kg KBr ha^−1^ to each of the lysimeters to provide information on the movement of water through the soil column. Lysimeters were cultivated with maize (9 plants per lysimeter, equally spaced). No fertilizers or treatments for weeding were executed during the study period.

### Sampling of leachates, soil, and plant material

Lysimeter soils were sampled from 0 to 5 cm depth (5 spots equally spaced in each lysimeter) at 4 dates over the study period (before and directly after application, 165 and 360 days after application). Soil sampling before application characterizes the basic level of GLYP*i* concentration, whereas the sample directly after application represents 100% of initial GLYP*i*. To keep the soil column intact, samples from the whole topsoil (0–30 cm) and the subsoil (30–60 cm) were taken only at the end (day 360 after application) of the study period. Soil samples were air dried and sieved (2 mm). Subsamples of the sieved soils were finely ground for further measurement with IR-MS. Residues of GLYP*i* and AMPA*i* were extracted from 5 g of the sieved soil in 40 mL of a 1 M KOH solution (shaking overnight and centrifugation for 10 min at 1558 g) and stored at − 20 °C until quantitation via HPLC-ESI-MS/MS.

Leachates were collected weekly in polyethylene canisters and volumes were recorded. Subsamples of 150 mL were taken and stored in a freezer at − 20 °C in 3 × 50 mL centrifuge tubes for further measurements with ion chromatography (IC) and HPLC-ESI-MS/MS. A total of 50 mL of each sample were lyophilized to dryness (− 50 °C, 0.025 mbar; Christ Alpha 1-4, Martin Christ Gefriertrocknungsanlagen GmbH, D-37250 Osterode, Germany) and solid residue amounts were weighed back and stored for measurements with isotopic ratio mass spectrometry (IR-MS).

Mature maize plants (roots, shoots, and cobs) were harvested in September 2017 from the two treated lysimeters and one untreated neighboring plot as reference. Subsamples of 3 plants per lysimeter were harvested for further measurements of plant biomass. Moist weight was determined followed by drying at 60 °C and measuring of dry matter weight. Plant compartment samples (root, shoot, and cobs) were shredded and subsequently finely ground separately and stored until further measurements with IR-MS.

### Sample analyses

#### Conservative tracer and isotope ratio analyses

Br^−^ tracer analysis in the leachate was performed using ion chromatography (column: Metrosep A SUPP 5150 × 4.0 mm, pre-column: Metrosep A SUPP 4/5 Guard, eluent: 0.3 mM Na_2_CO_3_ and 1.0 mM NaHCO_3_, flow: 0.7 mL min^−1^, separation mode: isocratic; Metrohm, D-70794 Filderstadt, Germany).

Isotopic ratios for ^15^N/^14^N and ^13^C/^12^C in soil, plant compartments, and lyophilized leachate samples were measured through the elemental analyzer (Eurovector EA, Via F.lli Cuzio 42, 27100 PAVIA, Italy; IR-MS GV-Isoprome, Elementar Analysensysteme GmbH, Elementar-Straße 1, 63505 Langenselbold, Germany) in the Institute for Nutritional Sciences, University of Gießen, Germany. For this purpose, finely ground soil and plant samples from the two treated sites and one untreated site (reference) were measured in triplicates. Lyophilized leachate samples from lysimeter leachates were measured in duplicates. Equations 1 and 2 show the calculation of δ^15^N and δ^13^C derived from isotopic ratios of the sample in relation to defined standard isotopic ratios from air for N and Pee Dee Belemnite (PDB) for C; values are generally given in ‰.

1 $$ {\delta}^{13}C=\left(\frac{{\left({}^{13}C/{}^{12}C\right)}_{\mathrm{sample}}}{{\left({}^{13}C/{}^{12}C\right)}_{\mathrm{PDB}}}-1\right) $$

2 $$ {\delta}^{15}N=\left(\frac{{\left({}^{15}N/{}^{14}N\right)}_{\mathrm{sample}}}{{\left({}^{15}N/{}^{14}N\right)}_{\mathrm{air}}}-1\right). $$

#### GLYP*i* and AMPA*i* analyses

Soil extracts and leachate samples were analyzed for GLYP*i* and AMPA*i* with HPLC-ESI-MS/MS after derivatization with fluorenylmethyloxycarbonyl chloride (FMOC-Cl), as described in Wirth et al. (2019). The utilized system was composed of an LC-2040C Nexera-i and a triple quadrupole mass spectrometer LCMS-8060 (Shimadzu, Duisburg, Germany) equipped with a heated ESI-source. The FMOC derivatives were separated on a Gemini 3 μm NX-C_18_ column (Column 1: 150 × 2 mm, Aschaffenburg, Phenomenex, Germany).

Non-isotope-labeled GLYP (LGC Standards, Wesel, Germany) was used as internal standard for GLYP*i* (Sigma Aldrich, Taufkirchen, Germany) quantitation. Since AMPA*i* is not commercially available as a standard substance, no HPLC-ESI-MS/MS-optimization and, thus, no calibration could be carried out for this compound. Therefore, AMPA*i* was determined only qualitatively. Analytes were detected in the multiple reaction monitoring (MRM) mode. The MRM transitions were determined and optimized utilizing standard compounds (Table 1). However, as AMPA*i* is not commercially available, instrumental MRM optimization for AMPA*i*-FMOC could not be performed. Therefore, the settings for the MRM transitions for this compound were chosen as follows: optimization was carried out for ^13^C-^15^N-AMPA-FMOC and AMPA-FMOC (LGC Standards, Wesel, Germany) and their fragmentation patterns were utilized to derive the expected masses of the precursor and product ions for ^15^N-AMPA-FMOC (AMPA*i*-FMOC). Further parameters of the MRM transitions were set by averaging values for ^13^C-^15^N-AMPA-FMOC and AMPA-FMOC (Table 1).

**Table 1:** Measurement modes for identification and quantitation of ^13^C_2_-^15^N-glyphosate and ^15^N-aminomethylphosphonic acid using high performance liquid chromatography tandem mass spectrometry (HPLC-ESI-MS/MS)

Component	Measurement mode	Precursor m/z	Product m/z	Collision energy	Retention time column 1(min)	Retention time column 2 (min)
^13^C_2_-^15^N-Glyphosate-FMOC (GLP*i*)	-	392.10	170.15	14	9.10	8.77
			152.20	24		
			63.10	48		
Glyphosate-FMOC	-	390.00	168.15	14	9.10	8.76
			150.20	23		
			63.05	49		
^15^N-AMPA-FMOC* (AMPA*i*)	+	335.20	179.05	− 23	9.47	9.18
			178.15	− 48		
			157.05	− 10		
			113.05	− 15		
AMPA-FMOC	+	334.20	179.05	− 23	9.47	9.19
			178.15	− 46		
			156.00	− 10		
			112.05	− 15		
^13^C-^15^N-AMPA-FMOC	+	336.20	179.05	− 22	9.46	n.a.
			178.10	− 50		
			158.15	− 10		
			114.10	− 15		

*derived from the optimized MRM transitions of ^13^C-^15^N-AMPA-FMOC and AMPA-FMOC

To further verify that the targeted and detected compound was the ^15^N-AMPA-FMOC, a selection of samples was additionally separated on a different LC-column (Column 2: Kinetex 2.6 μm EVO C18 100 Å, 150 × 2.1 mm, Phenomenex, Aschaffenburg, Germany). The proposed AMPA*i*-FMOC was eluted from both columns at similar retention times as AMPA-FMOC (Table 1) which confirms its presence. Due to the lack of an AMPA*i*-FMOC calibration, these data could be evaluated only semi-quantitatively. Quantitation of GLYP*i* was carried out through weighting with the glyphosate internal standard signal.

## Results and discussion

### Precipitation and leachate analysis

The study period from May 2017 to April 2018 was characterized by overall high amounts of precipitation that exceeded the monthly 30-year mean values (1981–2010) for this region, except for the months May, September, and February. Especially, June and July were characterized by heavy rainfall events that summed up to 123 and 125 mm per month precipitation, greatly exceeding the mean values of 57 ± 22 mm (June) and 61 ± 32 mm (July). These events resulted in large amounts of leachate in July 2017 (60.4 and 66.3 L). Weekly leachate amounts, collected from May 2017 until July 2017 to December 2017 until April 2018, had a mean volume of 5.1 L per week. For the period from August 2017 to November 2017, no leachates were received although precipitation occurred, most likely because of transpiration and water uptake by plants. Total volumes of leachates for the two lysimeters were 203 and 215 L over the study period. The Br^−^-breakthrough started in week 10 after application, where 35 and 37 L of leachate were received in the two tested lysimeters. Residues from the conservative tracer KBr were detected later on in all leachates. Due to the occurrence of Br^−^ in the leachates after 10 weeks and its slowly increasing concentrations over the following weeks along with continually received leachates, the main transport mechanism through the soil column can be assumed as matrix flow for the studied period.

**Fig. 1:**
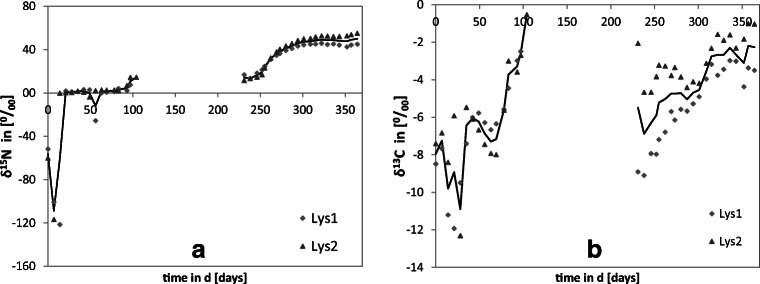
δ^15^N (**a**) and δ^13^C (**b**) values for lyophilized leachates over the one-year study period in lysimeter 1 (Lys1) and lysimeter 2 (Lys2) and mean values (continuous line)

For the natural ^15^N background representing the ratio of ^15^N/^14^N of the air nitrogen, the δ^15^N has been set to 0 (Fig. 1a). Discrepancies towards higher values indicate an enrichment of ^15^N. In the first 2 weeks after application, a strong decrease of leachate δ^15^N to negative values was detected, indicating an enrichment of ^14^N. In the following weeks 3 to 10, the δ^15^N in the leachate was constant between 0 and 1.7‰, and it increased over time from week 11 after GLYP*i* application. After the period with no leachates, the trend of δ^15^N had a sigmoidal shape with an assumed maximum limit of about 50‰ for the last 10 weeks of the experimental period. This maximum level corresponds to a mass rate of about 10 μg ^15^N week^−1^ of leached GLYP*i* active ingredient equivalent or its N-containing degradation products.

Values for δ^13^C started at about − 8‰ and fluctuated between − 12 and − 6‰ for the first 10 weeks before they strongly increased and reached values of − 2.6 and − 0.5‰ in the two lysimeters (Fig. 1b). After the period with no leachates, the δ^13^C started at lower levels of − 8.9 and − 2.1 in lysimeters 1 and 2, respectively. The trend of increasing δ^13^C values starting at − 5.5‰ went on and ended at −2.3‰ for the remaining 20 weeks of the study, although with a less steep slope than in the first experimental phase.

Trends of ^13^C and ^15^N originating from GLYP*i* in the leachate (Fig. 1) did not correlate, which may be an indication for an independent movement of these isotopes through the soil column. Also, IR-MS cannot distinguish between GLYP*i* and its degradation products, but simultaneous occurrence and parallel trend would be an indication for a displacement of intact GLYP*i*, which appears unlikely from these data. The analyses for GLYP*i* and AMPA*i* using HPLC-ESI-MS/MS in the leachates showed no occurrence of residues of these compounds above detection limits (0.1 μg L^−1^). Therefore, the leached ^15^N and ^13^C residues are most likely no constituents of intact GLYP*i* or AMPA*i* but originated from further degradation products.

### Soil analyses

**Fig. 2:**
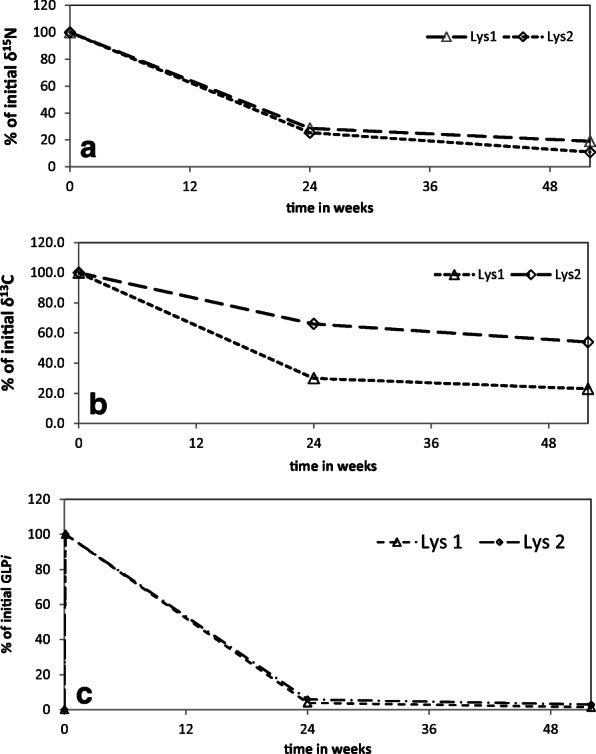
Development of δ^15^N (**a**), δ^13^C (**b**), and ^13^C_2_-^15^N-glyphosate (**c**) in topsoil samples compared with initial values (set to 100%) over the studied period in lysimeter 1 (Lys1) and lysimeter 2 (Lys2)

The concentrations of GLYP*i*, ^15^N and ^13^C in the lysimeter soils derived from HPLC-ESI-MS/MS and IR-MS, respectively, were normalized and set to 100% since GLYP*i* was not detectable in soil extracts sampled before GLYP*i* application (data not shown) (Fig. 2a). The δ^15^N decreased within 165 days after application to 24 and 29% of the initial value and decreased further to 11 and 19% until the end of the study period. This indicates that amounts of the added artificial ^15^N isotopes in soil decreased over time. The same was true for δ^13^C which decreased down to 30 and 66% compared with the initial value and ended at 23 and 54% in the two lysimeters (Fig. 2b).

Measurement of the GLYP*i* residues through HPLC-ESI-MS/MS showed that about 4 and 6% of the initial GLYP*i* concentration remained in the soil after 165 days and the recovery decreased further down to 1 and 3% in the two lysimeters until the end of the study (Fig. 2c). AMPA*i* was detected in the topsoil extracts of all samples after application (Suppl. Fig. S1), and GLYP*i* and AMPA*i* were not detected in the subsoil (results not shown). This indicates that AMPA*i* had not been formed, and GLYP*i* was already decomposed by microorganisms or scarcely displaced from surface into subsoil. Therefore, leaching of GLYP*i* or AMPA*i* can be considered as insignificant in this experiment and rapid degradation to further products most likely happened. This confirms Borggaard and Gimsing (2008) who reported a fast GLYP degradation and limited leaching through soil. Nevertheless, it is still possible that strongly bound non-extractable, and therefore non-detected residues of GLYP*i* or AMPA*i* could have remained in soil too, partly explaining the higher amounts of ^13^C and ^15^N after 165 and 360 days (Fig. 2a and b).

### Plant material analyses

**Fig. 3:**
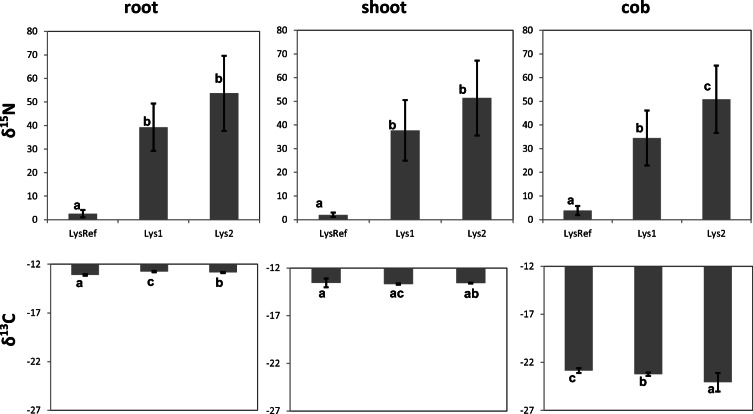
δ^15^N and δ^13^C mean values in plant material of roots, shoots, and cobs of maize plants of the tested lysimeter 1(Lys1), lysimeter 2 (Lys2), and a reference lysimeter (LysRef)

^15^N was enriched highly significantly (*p* < 0.01) in all sampled plant compartments (root, 39 ± 10‰ and 54 ± 16; shoot, 28 ± 13‰ and 51 ± 16‰; cob, 34 ± 12‰ and 51 ± 14‰) compared with reference plant parts from a lysimeter that was not treated with GLYP*i* (root, 2.5 ± 1.6‰; shoot, 2.0 ± 0.9‰; cob, 4.0 ± 1.9‰) (Fig. 3). By comparison, ^13^C was highly significantly enriched only in the plant roots from the two lysimeters treated with GLYP*i* (− 12.75 ± 0.07‰ and − 12.84 ± 0.06‰) compared with maize roots from the lysimeter with no herbicide treatment (− 13.03 ± 0.08‰). In contrast, ^13^C was significantly depleted (*p* < 0.01) in the cob material of plants from lysimeters with GLYP*i* treatment (− 23.24 ± 0.18‰ and − 24.06 ± 0.96‰) compared with those with no treatment (− 22.84 ± 0.25‰). There was not a significant difference in the shoots between the treated (− 13.69 ± 0.06‰ and − 13.58 ± 0.05‰) and non-treated lysimeters (− 13.56 ± 0.45‰).

**Fig. 4:**
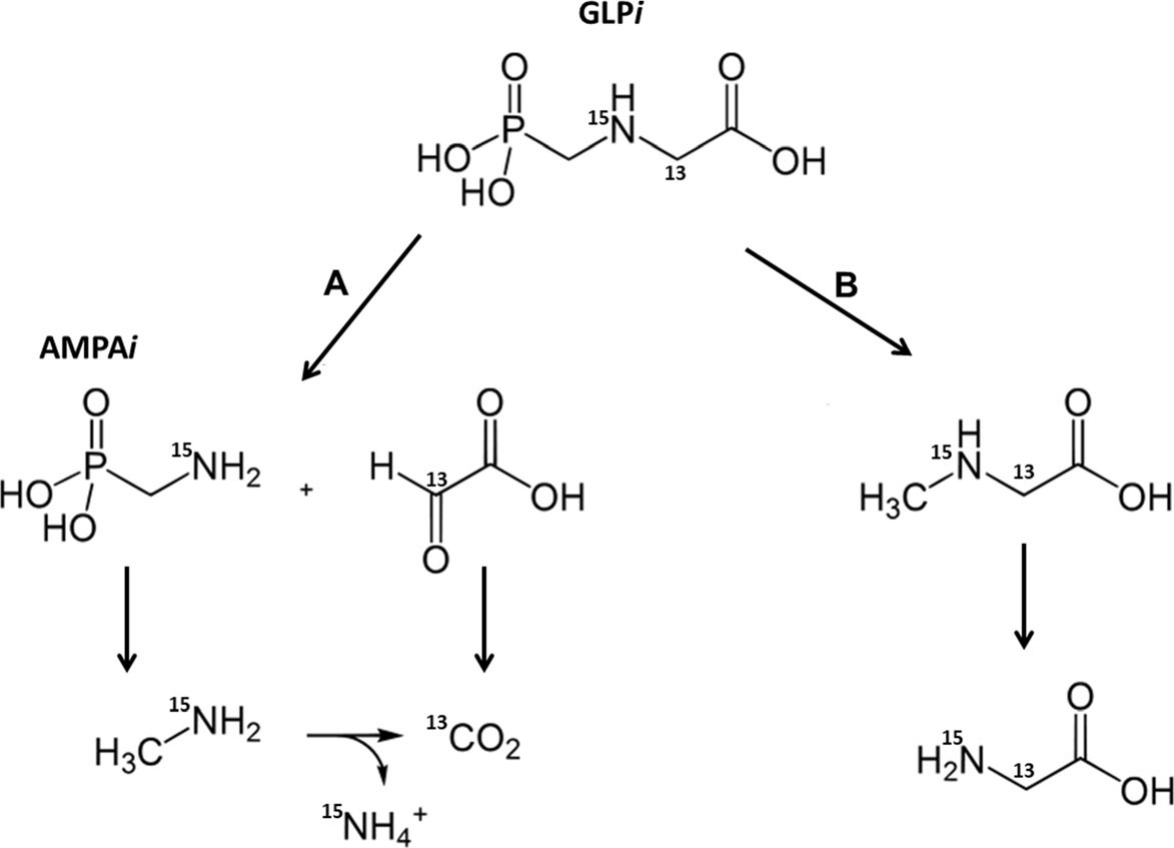
Degradation pathways of isotopic labeled ^13^C_2_-^15^N-glyphosate (GLP*i*) and its main degradation product ^15^N-aminomethylphosphonic acid (AMPA*i*) with indicated positions of labelling (modified from Giesy et al. 2000)

The enrichment of ^15^N in roots, shoots, and cobs can result only from uptake from the soil and distribution through the plant. Since ^15^N is bound in GLYP*i* or its ^15^N containing degradation products (Fig. 4), those degradation products must have acted as plant nutrients. Furthermore, as plants do not take up organic substances like GLYP*i* or AMPA*i* over the root system, the occurrence of ^15^N can be plausibly explained only by an uptake of mineral ^15^N (^15^NH_4_^+^ and/or ^15^NO_3_^−^) as mineralized degradation products from GLYP*i,* which are formed by microbial degradation in the rhizosphere (Duke et al. 2012).

The enrichment of ^13^C in the roots compared with plants from the non-treated lysimeter may either be a result of uptake or attachment to this plant compartment. The possibility of ^13^C uptake should be excluded, since neither organic substances like GLYP*i* or AMPA*i* can be taken up by plant roots, nor were these substances further distributed into other plant compartments. Also, mineralized species of ^13^C like ^13^CO_2_ originating from GLYP*i* degradation cannot be taken up by the roots. Therefore, enrichment of ^13^C species is explained by attachment, possibly due to mycorrhizal fungi associated with the maize roots (Bott et al. 2011) that utilize organic substances as nutrients for growth.

In summary, since (i) ^15^N has been taken up by the maize roots and distributed into all plant compartments and (ii) ^13^C is only associated with the plant roots, the interaction of these labeled atoms with the plants most plausibly resulted from the independent interaction of the inorganic degradation products of GLYP*i*. ^13^CO_2_ and ^15^NH_3_ as the inorganic end-products of the degradation process can be emitted via the air path. This was shown for ^14^C labeled GLYP (Grundmann et al. 2008). But since (i) ^15^NH_3_ is water-soluble and forms ^15^NH_4_^+^ in soil solution and (ii) ^15^N was taken up by plants, it is rather unlikely for inorganic N to be emitted into the air.

### Summarizing discussion

Comparing the present approach with an overview of most important published GLYP-lysimeter studies, the compilation in Table 2 shows that most experiments have been conducted with radioactive labelling of GLYP (*n* = 6) in the laboratory or with non-labeled GLYP (*n* = 3). Among the latter studies, only two reflected field conditions. Filtered leachates for GLYP residues have been analyzed in all studies, but only four (Al-Rajab et al. 2008; Grundmann et al. 2008; Klier et al. 2008; Bergström et al. 2011) additionally analyzed extracted GLYP residues or its degradation products in soil. Thus, the present study was designed in detail so that field conditions are reflected and also all relevant compartments are considered.

**Table 2:** Literature data of methods for lysimeter studies using glyphosate of different labelling

Reference	Country	Lysimeter soil column, site	Labelling	Application	Analytical detection	Studied compartment		
		(10^3^ cm^3^)		(kg ha^−1^)		Leachate	Soil	Plant
De Jonge et al. 2000	Denmark	8, laboratory	^14^C	2.4	Scintillation	x	x	
Fomsgaard et al. 2003	Denmark	550, laboratory	^14^C	0.8	GC-MS	x		
Dousset et al. 2004	France	3, laboratory	None	1.5	LC-ESI-MS/MS	x		
						x		
Malone et al. 2004	USA	19,440, field	None	0.5	FLD	x		
Kjær et al. 2005	Denmark	*(drained field*)	None	1.44	HPLC-EI-MS	x		
Al-Rajab et al. 2008	France	2, laboratory	^14^C	2.2	HPLC-FLD/RFD	x	x	
Grundmann et al. 2008	Germany	20, laboratory	^14^C	3 × 1	Scintillation, HPLC-RFD	x	x	x
Klier et al. 2008	Germany	20, laboratory	^14^C	10.8	Scintillation, HPLC-RFD	x	x	x
Bergström et al. 2011	Sweden	34, laboratory	^14^C	1.5	GC–MS	x	x	
present study	Germany	1000, field	^15^N-^13^C_2_	3.6	IR-MS, HPLC-ESI-MS/MS	x	x	x

*GC*, gas chromatography; *HPLC*, high performance liquid chromatography

*ESI-MS/MS*, electro spray ionization tandem mass spectrometry; *EI-MS*, electron impact mass spectrometry

*FLD*, fluorescence detection; *RFD*, radio flow detection

*IR-MS*, isotopic ratio mass spectrometry

In the present study, concentrations of extracted GLYP*i*-residues were low in soil at the end of the study period compared with the initial concentrations at the beginning. But fractions of ^15^N and ^13^C above extracted GLYP*i*-residues indicate that either non-extractable GLYP*i* is still left and/or further degradation products accumulated in soil. The latter explanation agrees with Al-Rajab et al. (2008), Grundmann et al. (2008), and Klier et al. (2008), who found low amounts of GLYP that remained in soil due to degradation of GLYP. Residues of GLYP and AMPA remained in topsoil (Bergström et al. 2011) and were either accumulated by organisms in the rhizosphere with low risk of leaching (Grundmann et al. 2008; Klier et al. 2008) or have been sorbed to soil particles with a risk of leaching (Al-Rajab et al. 2008).

The ^13^C and ^15^N are signals of leachates (Fig. 1), but absence or low concentrated (< LOD) residues of GLYP*i* and AMPA*i* indicate that further degradation products have been leached through the soil column, which partly confirms De Jonge et al. (2000), Fomsgaard et al. (2003), and Dousset et al. (2004). In contrast, leaching of GLYP and AMPA in lysimeters was reported for tilled soils and explained by particle transport (Fomsgaard et al. 2003; Malone et al. 2004; Kjær et al. 2005). This contradiction may result from different experimental designs, soil properties, and management measures that support or suppress particle-bound GLYP transport (Fomsgaard et al. 2003). Furthermore, the methods applied differ in their sensitivity to detect particle-bound GLYP or AMPA in leachates or soil extracts (Table 2). For instance, HPLC only provides information on the concentration of free or extractable amounts of GLYP or AMPA (Malone et al. 2004), since filtration is mandatory before HPLC-MS/MS measurements of liquid samples. However, HPLC cannot distinguish between bound (non-extractable) GLYP and further degradation products, since only free and recoverable GLYP can be detected. Most of the lysimeter studies complied in Table 2 used single ^14^C labelling (radioactive), which can be assigned to GLYP or AMPA in leachates or extracts by its retention time and molecular mass and/or radioactive signal when measured by HLPC in combination with mass spectrometry and/or scintillation detection. However, molecule identification only by scintillation of solid samples is not possible, since the signal of a labeled C-atom originate come from intact GLYP or degradation products, even if the position of the labelling in the initial GLYP molecule is known. Therefore, the multi-labelling of GLYP with ^15^N and ^13^C in the present field lysimeter study, by avoiding disadvantages of previous studies, has shown (time-) independent occurrence of GLYP*i* decomposition products. The noncorrelated appearance of ^13^C and ^15^N signals in leachates (Fig. 1) makes the degradation pathway B in Fig. 4 rather unlikely, in agreement with Borggaard and Gimsing (2008). Instead, pathway A in Fig. 4 is supported by the noncorrelated appearance of ^13^C and ^15^N signals in leachates, among which ^13^C can originate from glyoxylic acid and ^15^N from detected AMPA*i* or further degradation products, such as methylamine and ammonium ions (Fig. 4). Along with small concentrations of extracted GLYP*i* (Fig. 2c) under practically optimal leaching conditions in the very wet hydrological year 2017/2018, the present findings indicate that rapid degradation most likely is the best explanation for the absence of concentrations below LOD of GLYP*i* and AMPA*i* in leachates, which confirms previous findings as referenced in Table 2.

## Conclusions

Isotopic ratios of ^13^C/^12^C and ^15^N/^14^N and resulting changes of δ^13^C and δ^15^N from isotopically labeled glyphosate (GLYP*i*) and its degradation products were successfully quantified using isotopic ratio mass spectrometry (IR-MS) in different compartments (leachates, soil, and plant material) of a field lysimeter. Therefore, this experimental approach was well suited to trace GLYP*i* under practice-near experimental conditions.

Since (i) the great decline of GLYP*i* content down to < 3% of initial amounts in soil during the one-year study period and (ii) a lower decline of ^13^C (< 60%) and ^15^N (< 20%), we conclude that either further degradation products had been formed and/or non-extractable and, therefore, strongly bound GLYP*i* remained in soil and accumulated. The disparate increase of δ^13^C and δ^15^N values in leachates and plant material is explained plausibly by (i) rapid degradation of GLYP*i* within one vegetation period and, also (ii) the selective uptake of mineralized ^15^N species from degraded GLYP*i* as plant nutrient, most likely NH_4_^+^ or NO_3_^−^. These findings from a wet hydrological year support the assumption that the risk of leaching of applied GLYP to other waterbodies can be considered to be low under central European climatic conditions. Accumulation in soil may enhance the risk of further distribution in the environment by soil erosion.

## Electronic supplementary material

Figure S1 Chromatograms of the standard substance aminomethylphosphonic acid (AMPA, dotted line, transition 334.2 *m/z* -> 178.15 *m/z*) from analytical standard sample and ^15^N-aminomethylphosphonic acid (AMPA*i*, straight line, transition 335.2 *m/z* -> 178.15 *m/z*) from a topsoil sample extract for identification (retention time 9.47 min, vertical dashed line. (DOCX 21 kb)
