# Supplementary material for: Leaching and degradation of 13C2-15N-glyphosate in field lysimeters
Source: Environ Monit Assess. 2020 Jan 21;192(2):127. doi: 10.1007/s10661-019-8045-4 (PMC6970956; doi:10.1007/s10661-019-8045-4)
Supplement: Supplementary file 1 — Chromatograms of the standard substance aminomethylphosphonic acid (AMPA, dotted line, transition 334.2 m/z -> 178.15 m/z) from analytical standard sample and 15N-aminomethylphosphonic acid (AMPAi, straight line, transition 335.2 m/z -> 178.15 m/z) from a topsoil sample extract for identification (retention time 9.47 min, vertical dashed line. (DOCX 21 kb) [file 10661_2019_8045_MOESM1_ESM.docx]

Supplementary

Figure S1: Gros *et al.*, 2019
